# Power-Dependent Optical Characterization of the InGaN/GaN-Based Micro-Light-Emitting-Diode (LED) in High Spatial Resolution

**DOI:** 10.3390/nano13132014

**Published:** 2023-07-06

**Authors:** Haifeng Yang, Yufeng Li, Jiawei Wang, Aixing Li, Kun Li, Chuangcheng Xu, Minyan Zhang, Zhenhuan Tian, Qiang Li, Feng Yun

**Affiliations:** 1 Shaanxi Provincial Key Laboratory of Photonics & Information Technology, Xi’an Jiaotong University, Xi’an 710049, China; yhfeng@stu.xjtu.edu.cn (H.Y.); wangjiawei199971@stu.xjtu.edu.cn (J.W.); liaixing@stu.xjtu.edu.cn (A.L.); likun2018@xjtu.edu.cn (K.L.); xcc1025@stu.xjtu.edu.cn (C.X.); zhangmy@xjtu.edu.cn (M.Z.); tianzhenhuan@mail.xjtu.edu.cn (Z.T.); liqiang@mail.xjtu.edu.cn (Q.L.); 2 Solid-State Lighting Engineering Research Center, Xi’an Jiaotong University, Xi’an 710049, China

**Keywords:** micro-LED, sidewall damage, spatially resolved photoluminescence

## Abstract

Spatially resolved photoluminescence at the sub-micro scale was used to study the optical non-uniformity of the micro-LED under varied power density excitation levels. The trend of the efficiency along injection levels were found to be highly dependent on the location of the chip mesa. The sidewall was 80% lower than the center under low-power density excitation, but was 50% higher under high-power density excitation. The external quantum efficiency droop at the center and the sidewall was 86% and 52%, respectively. A 2 µm band area near the sidewall was characterized where the efficiency and its trends changed rapidly. Beyond such band, the full width at half maximum and peak wavelength variation across the chip varied less than 1 nm, indicating high uniformity of the material composition. The sudden change = in the band, especially under high level excitation indicates the indium composition change formed by ion residues on the sidewall affect the distribution of charge carriers. These findings contribute to the understanding of cause of efficiency disadvantage and non-uniformity problems in small-size micro-LEDs.

## 1. Introduction

The GaN-based micro-light-emitting diode (micro-LED) shows excellent performance in brightness, electro-optical conversion efficiency, lifetime and response time [1,2,3,4], which has significant potential in the areas of advanced display technology, optogenetics and visible light communication (VLC). Micro-LEDs have higher resolution, better current distribution and heat dissipation. However, the decreased chip size presents a number of difficulties, such as more serious Shockley-Read-Hall (SHR) recombination and lower external quantum efficiency (EQE), which is caused by the defects at the edges [5,6]. The commercialization of high-efficiency small-scale micro-LEDs (smaller than 20 × 20 µm) is limited by potential damage at the sidewalls of the chip, such that intense focuse has been put on the influence of sidewall damage of small-sized micro-LEDs. Due to the reduction in the size of micro-LEDs, specialized optical characterization techniques have been realized, such as micro-photoluminescence (micro-PL) [7], laser confocal scanning microscope (LCSM) [8], electroluminescence spectroscopy (EL) [9] and cathodoluminescence spectroscopy (CL) [10]. Fan Yang et al. found that the PL intensity near the sidewall of small-sized micro-LEDs was reduced due to the presence of etching damage [11]. They claimed that this phenomenon was caused by the sidewall damage. The PL intensity could be improved by combining SiO_2_ passivation and tetramethylammonium hydroxide treatment. Additionally, the uniformity of PL intensity was greatly improved through this treatment. Olivier François et al. found that the luminescence intensity near the sidewall was higher than that in the middle by imaging in a charge coupled device (CCD) [12]. They showed that smaller micro-LEDs could provide more significant brightness near the sidewalls. They explained that such brightness was due to the leakage channel in the sidewall. Leakage channels were caused by the ionic compounds left over from the etching process, which reduced the equivalent impedance of the edge. The current in small mesa could easily diffuse to the edge; thus, the luminescence was enhanced near the sidewall of micro-LEDs with smaller sizes. Such two contradictory observations are not unique, but seem to appear repeatedly among other studies [13,14,15,16,17,18,19,20]. However, these studies did not give an explanation for this non-uniform luminescence under uniform injection of carriers (PL), and whether it was related to the excitation power density. The dominant factors that cause the efficiency droop in micro-LEDs under low-level power density and high-level power density excitation are different. Therefore, a characterization method combining high spatial resolution and a high-power density injection range is necessary to confirm the mechanism and its relationship to the damage in micro-LEDs.

In our work, a micro-characterization method which combines high spatial resolution and a large scale of power density variation was used to record PL intensity mapping, full width at half height (FWHM) and peak wavelength distribution of micro-LEDs under different power densities. EQE droop was adopted to accurately define the influence range of sidewall damage, providing a reference for micro-LED manufacturing and sidewall treatment.

## 2. Materials and Methods

**Figure 1:**
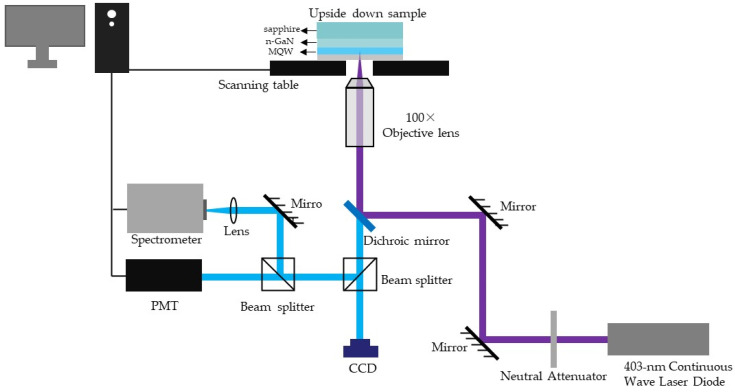
Schematic of scanning micro-photoluminescence measurement system.

The blue LED epitaxial wafer was grown via metal-organic compound vapor deposition (MOCVD). The epitaxial structure consisted of a 2 µm thick GaN nucleation layer, along with a 2 µm think Si-doped n-GaN, 12 periods of InGaN/GaN MQWs and 240 nm thick Mg-doped p-GaN. micro-LED mesa arrays were fabricated to square mesas with a size of 20 × 20 µm^2^ by inductively coupled plasma (ICP) etching with the etching gas of Cl_2_/BCl_3_ (see Figure S1). Figure 1 shows the schematic of a scanning micro-photoluminescence measurement system. A continuous-wave (CW) laser diode (Power Technology, Alexander, AR, USA) with excitation wavelength 403 nm was used as the laser source. The laser was focused through a 100× lens with NA = 0.9 from Olympus Inc. The focused size of the laser spot was 900 nm and a neutral attenuator was used to control the energy density of the injected laser. The injected laser power density ranged from 5.8 × 10^3^ to 7.6 × 10^6^ W/cm^2^. A CCD was used to ensure the accuracy of the optical path of the test system. The PL signal was collected by the same objective lens and was directed to a photo multiplier tube (PMT) for PL mapping after passing through a 413 nm long-pass filter. The 413 nm long-pass filter was used to filter the laser reflected by the sample. A diffraction grating spectrometer (HORIBA-iHR550, Jobin Yvon, Kyoto, Japan) with an optical resolution of 0.02 nm was used to capture spectral signals from samples. A temperature control stage (THMS600, Linkam, Salfords, UK) was used to cool down the sample. An optical table with an air floating vibration isolation was used to install the whole installation. The sample was mounted on an xy-axis piezo nano displacement scanner with a resolution of 0.1 nm, which was a part of near-field optical microscopy (SNOM). SNOM (NTEGRA Solaris Probe NanoLaboratory produced by NT-MDT, Moscow, Russia) was used to study near-surface optical characteristics (including luminescent and spectral characteristics) of various objects with resolution much greater than the diffraction limit. The higher resolution was gained by exposing samples to light going through a diaphragm whose aperture was much less than the wavelength of the radiation detected. The surface under study lies in the near field of the diaphragm (at distances of about 10 nm).

A 403 nm CW laser beam was injected from the p-GaN, which was 240 nm in the sample. It was assumed that the laser was majorly absorbed by the active layer and the excited carries were confined in the quantum well, with high radiation composite efficiency [21,22]. The transport processes of carriers in barriers and other parts of the MQWs along with the leakage currents can be neglected. Unlike the non-uniform current spreading under EL injection, the distribution of carries was uniform across the whole micro-LED under PL excitation (see Figure S2).

## 3. Results and Discussion

**Figure 2:**
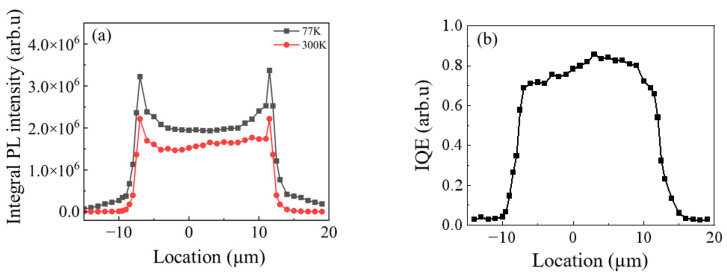
(**a**) Integral PL intensity under laser power density of 7.6 × 10^6^ W/cm^2^ as a function of position at 77 K and 300 K. (**b**) The IQE extracted from Figure 2a.

The PL intensity across the micro-LED was measured at room temperature (RT) and at 77 K under an injection of 7.6 × 10^6^ W/cm^2^ (Figure 2a). The internal quantum efficiency (IQE) at RT was calculated by the ratio of I_(RT)_/I_(77K)_. Figure 2b shows that IQE was high in the center and was much lower near the sidewall. One can estimate 80% of the IQE drop within approximately 1 µm near the sidewall. Similar degradation was observed for AlGaInP-based micro-LEDs, which is attributed to long carrier diffusion length in AlGaInP [23]. The relationship between integral PL intensity and IQE is that:

(1) $$I_{\mathrm{PL}}\propto \eta_{\mathrm{EQE}}\times \eta_{\mathrm{LEE}}$$

where I_PL_ denotes the integral PL intensity. By comparing the I_PL_ and IQE at the sidewall and the center, we found that the light extraction efficiency (LEE) at the edge was 68% higher than that at the center. Thus, the non-uniform luminescence under the high excitation region was caused by a higher LEE at the sidewalls.

**Figure 3:**
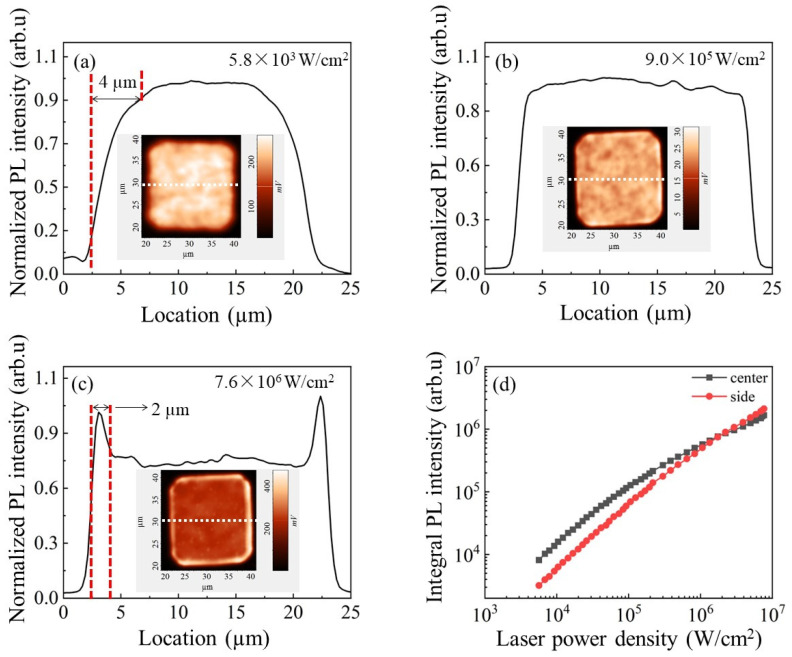
(**a**–**c**) Normalized PL intensity mapping pictures of the same pixel for different excitation laser power densities: intensity level (black line) is extracted from the inset (white dashed line). (**d**) Integral PL intensity at the sidewall at the center of this pixel as a function of the excitation laser power density.

Micro-PL intensity mapping was carried out at different excitation power densities. Figure 3a–c show the PL intensity mapping under of 5.8 × 10^3^ W/cm^2^, 9 × 10^5^ W/cm^2^ and 7.6 × 10^6^ W/cm^2^, respectively. The PL intensity dropped 75% from the center to the sidewalls under 5.8 × 10^3^ W/cm^2^ excitation within 4 µm from the edge. Such degradation disappeared under 9 × 10^5^ W/cm^2^ and the PL intensity remained almost unaffected across the chip. Only when the distance from the laser spot to the edge was less than 1 µm did the PL intensity decrease significantly. When the excitation power reached 7.6 × 10^6^ W/cm^2^, the PL intensity near the sidewalls was 40% higher than that at the center and the “improved area” was measured in a 2 μm band close to the etched surface. This uneven luminescence was observed with untreated sidewalls. The degradation of PL intensity under low-level power density excitation was attributed to a stronger non-radiative recombination process at the sidewalls. A similar phenomenon was found in previous research and the improved luminescence under high-level power density excitation was explained by the uneven current spreading caused by the smaller equivalent resistance at the sidewall [12]. Figure 3d shows the PL intensity as a function of the excitation power density at the center and sidewall. The integrated PL intensity was higher at the center from 5.8 × 10^3^ W/cm^2^ to 1 × 10^6^ W/cm^2^, due to the Shockley-Read-Hall (SHR) recombination from edge defects [11]. The PL emission became more efficient at the sidewall from 1 × 10^6^ W/cm^2^ to 7.6 × 10^6^ W/cm^2^, probably enhanced by the modified LEE of the sidewall [24]. The LEE at other regions of the micro-LED was lower than that at the sidewalls due to the total internal reflection.

**Figure 4:**
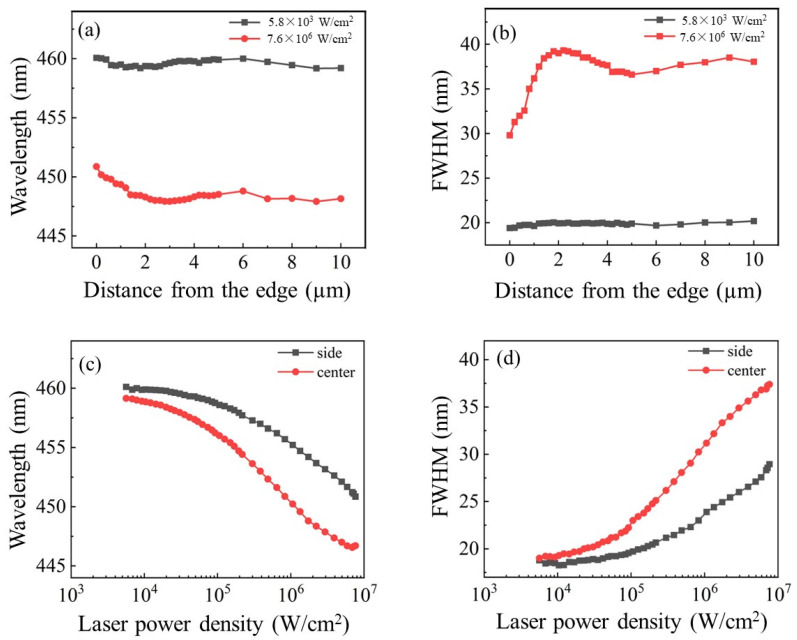
(**a**) The wavelength at the side and the center as a function of excitation laser power density, (**b**) FWHM at the side and the center as a function of excitation laser power density. (**c**) FWHM as a function of distance from the edge under 5.8 × 10^3^ W/cm^2^ and 7.6 × 10^6^ W/cm^2^, (**d**) corresponding peak wavelength as a function of distance from the edge.

Figure 4a,b show the wavelength and full width at half height (FWHM) at the side and center of the micro-LED as a function of excitation power density. When the excitation power density increased from 5.8 × 10^3^ W/cm^2^ to 7.6 × 10^6^ W/cm^2^, the peak wavelength near the sidewall blue-shifted from 460.1 to 450.8 nm and it shifted from 459.2 to 448.1 nm (see Figure S3) in the center due to the screen effect of the Quantum-Confined Stark Effect [25]. The FWHMs of the spectra were 19.4 and 20.2 nm for the side and center, respectively, at 5.8 × 10^3^ W/cm^2^, and they increased up to 29.8 and 38 nm at 7.6 × 10^6^ W/cm^2^ (see Figure S4). Such widening illustrates the significance of the band filling effect at the center of the micro-LED, resulting in a worse EQE droop.

Figure 4c,d show the peak wavelength and FWHM as a function of distance from the edge under extremely high and low excitation levels. The peak wavelength across the mesa varied little from 459.18 nm to 460.07 nm under low levels of excitation. The variation was also stable under high levels of excitation except for the 2 μm band close to the etched surface. Figure 4d shows that the FWHM varied a little from 19.40 nm to 20.20 nm under low levels of excitation. Sudden decreases in FWHM were found in the 2 μm band area under high levels of excitation. The reason for the FWHM shrinkage near the sidewall under high power density excitation is that the indium composition near the sidewalls was changed by the etching process, which affected the distribution of the charge carriers [11].

Power-dependent PL measurements were carried out from the side to the center of a 20 × 20 µm^2^ mesa, and PL external quantum efficiency (EQE) was estimated by [26]:

(2) $$\eta_{\mathrm{EQE}}=C\frac{I_{\mathrm{PL}}/E_{\mathrm{PL}}}{I_{\mathrm{EX}}/E_{\mathrm{EX}}}$$

where C is a constant related to the light absorption by the QWs; I_EX_ is the excitation optical power density; E_PL_ is the emitted photon energy; and E_EX_ is the laser photon energy.

**Figure 5:**
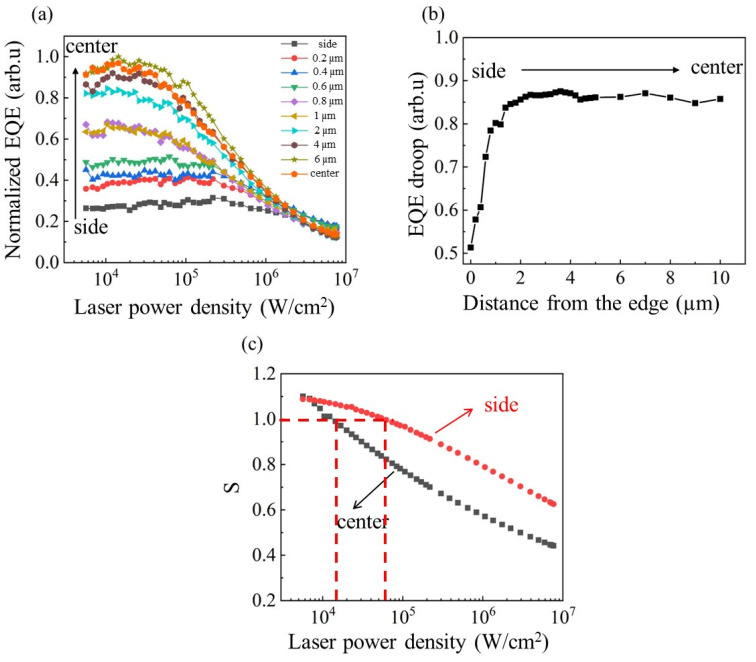
(**a**) Normalized EQE curves at different positions of micro-LEDs (chip size: 20 × 20 µm^2^) as a function of laser power density. (**b**) EQE droop curve as a function of distance from the edge. (**c**) S factor at side and center as a function of laser power density.

Figure 5a exhibits the normalized EQE curve across the chip. At high-level excitation densities, the EQE near the sidewalls was higher than other positions. While at low current densities, the EQE shows the opposite behavior due to the SRH non-radiative recombination. The excitation density of the maximum EQE near the mesa edge was estimated about 4.5 × 10^4^ W/cm^2^, and the value near the center was 10 times smaller, indicating a different EQE droop mechanism.

The EQE droop was used to estimate the damage area, which was defined as:

(3) $$Q_{\mathrm{droop}}=\frac{\eta_{\mathrm{EQE}}^{\max}-\eta_{\mathrm{EQE}}^{\max\mathrm{power}\;}}{\eta_{\mathrm{EQE}}^{\max}}$$

where $\eta_{\mathrm{EQE}}^{\max}$ is the max of the EQE, $\eta_{\mathrm{EQE}}^{\max\;\mathrm{power}}$ on behalf of the max excitation power EQE value.

Figure 5b shows the EQE droop as a function of the distance from the sidewall. Given the optical resonant excitation, the carrier injection efficiency was assumed spatially independent. the EQE droop was found to become larger towards the chip center. Several reports have shown that micro-LEDs with larger sizes have better peak EQEs, but also have larger EQE droops than smaller ones [27,28,29,30,31,32,33,34]. They attribute this phenomenon to the sidewall damage (dead zone) [35,36]. Our experimental data show that the “dead zone” was less than 800 nm, compared to 180 nm in the research of Luming Yu et al. [35]. The EQE droop curve of the large-size micro-LED was more consistent with the curve in the center. As the size decreased, the EQE droop curve became more similar to the sidewall curve, which means that in micro-LEDs of small size, the efficiency is dominated by the sidewalls. 

Figure 5c exhibits the variation of the S factor in the center and side of the mesa, which is defined as ∂log(I_PL_)/∂log(P), as a function of excitation power density [37], and the PL intensity can be described as:

(4) $$I_{\mathrm{PL}}=\eta_{\mathrm{LEE}}{\mathrm{kBn}}^{2}$$

where B is the radiative recombination coefficient, n is the carrier concentration in multi-quantum well region, and k is a constant affected by the experiment system and the sample itself, does not depend on either injection power density or the position of measurement [38,39].

The S factor will converge to a value smaller than one; if the Auger recombination is a dominant carrier loss mechanism, an S factor of one is related to the radiative recombination. Meanwhile, an S factor bigger than one is mainly caused by the non-radiative recombination by defects [37]. It can be observed in Figure 5c that the S factor is decreasing with excitation power increasing at the side and center, and the S factor falls faster at the center. The level of defect saturation increased along with a higher injection of laser power density at steady-state injection [40]. There was a higher power density at S = 1 near the sidewall, implying a higher defect density at the sidewall. When the laser power density was higher than 5 × 10^4^ W/cm^2^, the S factor at the sidewall was bigger than the center ones, indicating that the Auger recombination was more remarkable at the center and, thus, a bigger EQE droop occurred at the center.

## 4. Conclusions

In conclusion, our comprehensive investigation of micro-LEDs under variable laser power density excitation revealed significant differences in PL intensity, internal and external efficiency, peak wavelength, and FWHM between the sidewall and center. Under low excitation, the PL intensity near the sidewall was only 20% of the center, but this increased to 150% under high excitation due to carrier diffusion and defect saturation. Notably, the FWHM and peak wavelength variations were less than 1 nm under low excitation, while high laser power density injection caused a distinct band shift attributed to indium composition changes from the ICP process, influencing charge carrier distribution. Our study highlights the sidewalls’ dominance in micro-LED efficiency, particularly in small-sized devices. Furthermore, we quantified the EQE droop under varying power excitation and identified a wide etching damage range of up to 800 nm using EQE droops at various sites. These findings underscore the valuable applications of high spatial resolution and high power density injection methods for quantitative measurements of micro-LED PL spectra and local EQE variations. By offering insights into optimizing efficiency and addressing sidewall damage, our research serves as a vital guide for enhancing the performance of small size micro-LEDs.

## Data Availability

Data underlying the results presented in this paper are not publicly available at this time, but may be obtained from the authors upon reasonable request.

## Supplementary Materials

The following supporting information can be downloaded at: https://www.mdpi.com/article/10.3390/nano13132014/s1, Figure S1: SEM picture of 20 × 20 µm^2^ Micro-LED, Figure S2: Image of a Micro-LED under laser excitation laser spot at (a) center, (b) side, and (c) corner. The scale of the solid white line in the figure is 5 µm, Figure S3: PL spectrum of Micro-LED at side and center at a laser power density of (a) 7.6 MW/cm^2^ and (b) under 5.8 kW/cm^2^, Figure S4: PL spectra of Micro-LED under different excitation power density at the (a) sidewall, and (b) center. PL spectra at different positions from center to the sidewall under (c) 7.6 × 106 W/cm^2^ and (d) 5.8 × 103 W/cm^2^.
