# Supplementary material for: Power-Dependent Optical Characterization of the InGaN/GaN-Based Micro-Light-Emitting-Diode (LED) in High Spatial Resolution
Source: Nanomaterials (Basel). 2023 Jul 6;13(13):2014. doi: 10.3390/nano13132014 (PMC10343276; doi:10.3390/nano13132014)
Supplement: Supplementary file 1 [file nanomaterials-13-02014-s001.zip › nanomaterials-2459675-supplementary.pdf]

Supplementary Information

Power-Dependent Optical Characterization of the InGaN/GaN-Based Micro-Light-Emitting-Diode (LED) in High Spatial Resolution

Haifeng Yang [1,2], Yufeng Li [1,2,*], Jiawei Wang [1,2], Aixing Li [1,2], Kun Li [1,2], Chuangcheng Xu [1,2], Minyan Zhang [1,2], Zhenhuan Tian [1,2], Qiang Li [1,2] and Feng Yun [1,2,*]

1 Shaanxi Provincial Key Laboratory of Photonics & Information Technology, Xi'an Jiaotong University, Xi'an 710049, China; yhfeng@stu.xjtu.edu.cn (H.Y.); wangjiawei199971@stu.xjtu.edu.cn (J.W.); liaixing@stu.xjtu.edu.cn (A.L.); likun2018@xjtu.edu.cn (K.L.); xcc1025@stu.xjtu.edu.cn (C.X.); zhangmy@xjtu.edu.cn (M.Z.); tianzhenhuan@mail.xjtu.edu.cn (Z.T.); liqiang@mail.xjtu.edu.cn (Q.L.)
2 Solid-State Lighting Engineering Research Center, Xi'an Jiaotong University, Xi'an 710049, China
* Correspondence: yufengli@mail.xjtu.edu.cn (Y.L.); fyun2010@mail.xjtu.edu.cn (F.Y.)

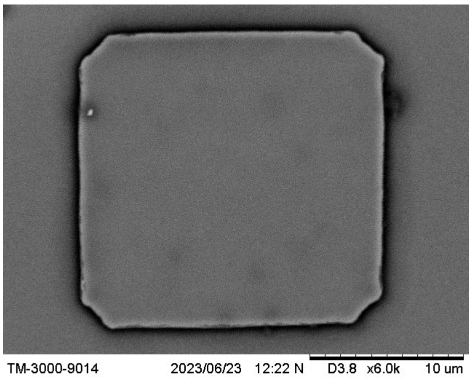

Figure S1. SEM picture of 20×20μm² Micro-LED.

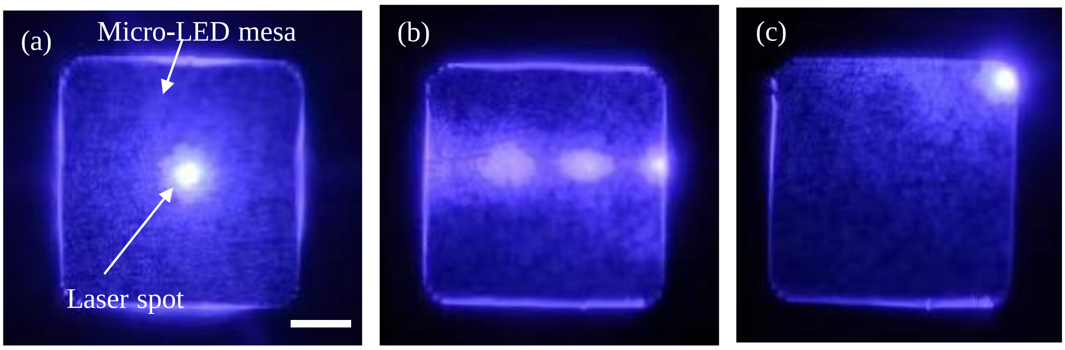

Figure S2. Image of a Micro-LED under laser excitation laser spot at (a) center, (b) side, and (c) corner. The scale of the solid white line in the figure is 5 μm.

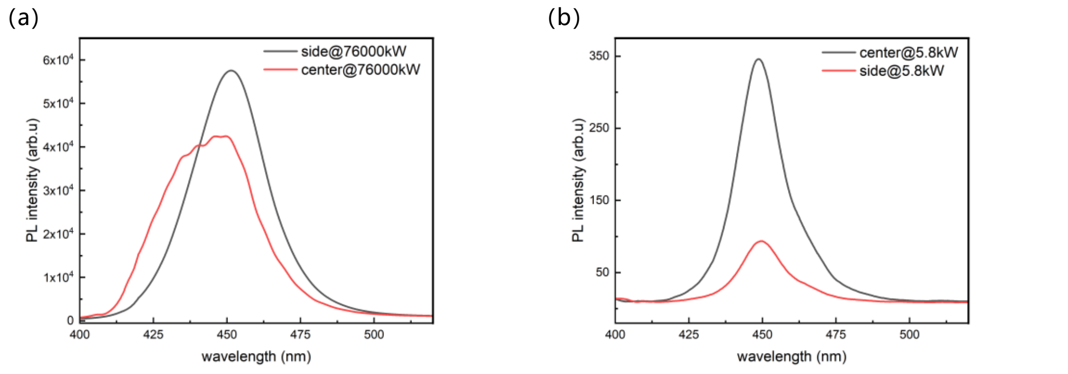

Figure S3. PL spectrum of Micro-LED at side and center at a laser power density of (a) 7.6MW/cm² and (b) under 5.8kW/cm².

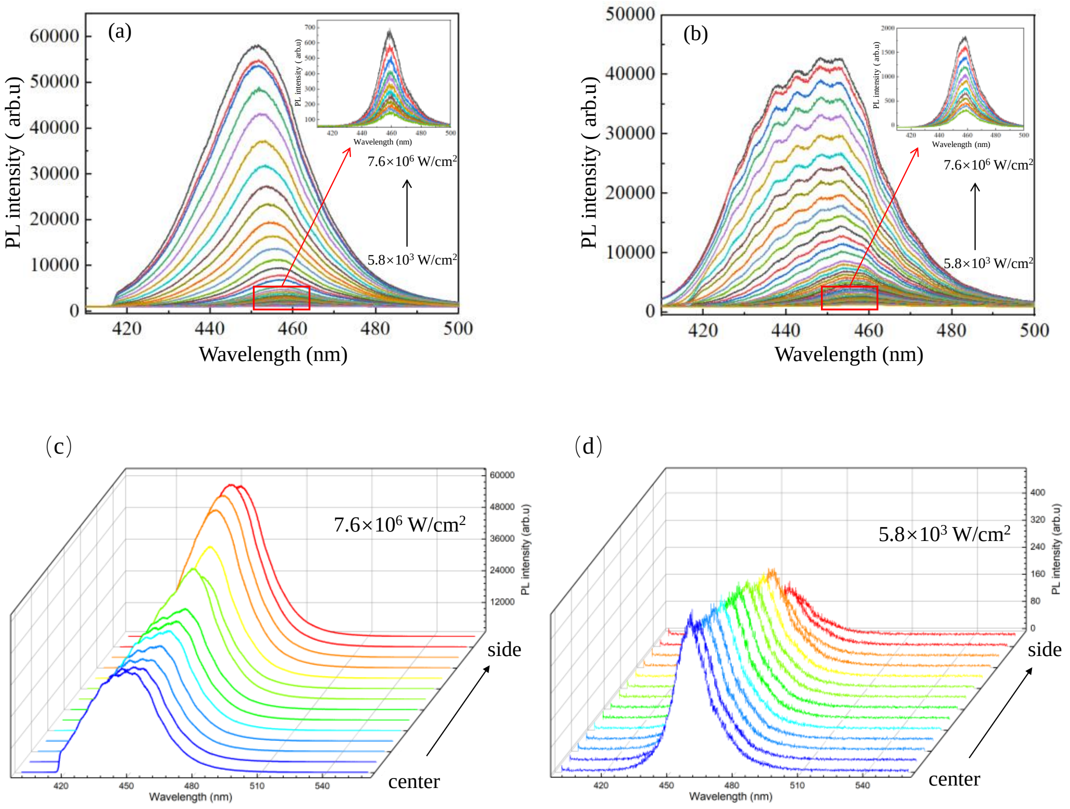

Figure S4. PL spectra of Micro-LED under different excitation power density at the (a) sidewall, and (b) center. PL spectra at different positions from center to the sidewall under (c) 7.6×10^6 W/cm^2 and (d) 5.8×10^3 W/cm^2.
